# Cost-Effectiveness of Coronary Artery Calcium Testing for Coronary Heart and Cardiovascular Disease Risk Prediction to Guide Statin Allocation: The Multi-Ethnic Study of Atherosclerosis (MESA)

**DOI:** 10.1371/journal.pone.0116377

**Published:** 2015-03-18

**Authors:** Eric T. Roberts, Aaron Horne, Seth S. Martin, Michael J. Blaha, Ron Blankstein, Matthew J. Budoff, Christopher Sibley, Joseph F. Polak, Kevin D. Frick, Roger S. Blumenthal, Khurram Nasir

**Affiliations:** 1 Johns Hopkins Bloomberg School of Public Health, Department of Health Policy and Management, Baltimore, Maryland, United States of America; 2 Johns Hopkins University School of Medicine, Ciccarone Center for the Prevention of Heart Disease, Baltimore, Maryland, United States of America; 3 Brigham and Women's Hospital, Cardiovascular Division, Boston, Massachusetts, United States of America; 4 UCLA School of Medicine, Department of Cardiology, Los Angeles, California, United States of America; 5 Knight Cardiovascular Institute, Oregon Health & Science University, Portland, OR, United States of America; 6 Tufts University School of Medicine, Lemuel Shattuck Hospital, Boston, Massachusetts, United States of America; 7 Johns Hopkins Carey Business School and Johns Hopkins Bloomberg School of Public Health, Baltimore, Maryland, United States of America; 8 Center for Healthcare Advancement and Outcomes, Baptist Health South Florida, Miami, FL, United States of America; 9 Miami Cardiovascular Institute (MCVI), Baptist Health South Florida, Miami, FL, United States of America; 10 Department of Epidemiology, Robert Stempel College of Public Health, Florida International University, Miami, FL, United States of America; University Medical Center Rotterdam, NETHERLANDS

## Abstract

**Background:**

The Multi-Ethnic Study of Atherosclerosis (MESA) showed that the addition of coronary artery calcium (CAC) to traditional risk factors improves risk classification, particularly in intermediate risk asymptomatic patients with LDL cholesterol levels <160 mg/dL. However, the cost-effectiveness of incorporating CAC into treatment decision rules has yet to be clearly delineated.

**Objective:**

To model the cost-effectiveness of CAC for cardiovascular risk stratification in asymptomatic, intermediate risk patients not taking a statin. Treatment based on CAC was compared to (1) treatment of all intermediate-risk patients, and (2) treatment on the basis of United States guidelines.

**Methods:**

We developed a Markov model of first coronary heart disease (CHD) and cardiovascular disease (CVD) events. We modeled statin treatment in intermediate risk patients with CAC≥1 and CAC≥100, with different intensities of statins based on the CAC score. We compared these CAC-based treatment strategies to a “treat all” strategy and to treatment according to the Adult Treatment Panel III (ATP III) guidelines. Clinical and economic outcomes were modeled over both five- and ten-year time horizons. Outcomes consisted of CHD and CVD events and Quality-Adjusted Life Years (QALYs). Sensitivity analyses considered the effect of higher event rates, different CAC and statin costs, indirect costs, and re-scanning patients with incidentalomas.

**Results:**

We project that it is both cost-saving and more effective to scan intermediate-risk patients for CAC and to treat those with CAC≥1, compared to treatment based on established risk-assessment guidelines. Treating patients with CAC≥100 is also preferred to existing guidelines when we account for statin side effects and the disutility of statin use.

**Conclusion:**

Compared to the alternatives we assessed, CAC testing is both effective and cost saving as a risk-stratification tool, particularly if there are adverse effects of long-term statin use. CAC may enable providers to better tailor preventive therapy to patients' risks of CVD.

## Introduction

Atherosclerotic cardiovascular disease (CVD), including coronary heart disease (CHD) and stroke, costs the United States an estimated $315 billion annually [1]. Cardiologists rely heavily on risk prediction models to identify and treat patients who are at risk for CVD events [2–7]. However, heterogeneity between traditional risk factors, subclinical atherosclerosis, and clinical outcomes is well documented [8,9]. This discordance is greatest among patients classified as “intermediate-risk”, and this has motivated a debate about whether better markers of risk are needed to guide treatment, or alternatively, whether universal treatment with statins is preferred, in light of the growing availability of low-cost generic statins [10]. Coronary artery calcium (CAC) measurement has proven useful for prognostication, discrimination, calibration, and reclassification for CHD and CVD. Studies such as the Multi-Ethnic Study of Atherosclerosis (MESA) and the Heinz Nixdorf Recall (NHR) Study have shown that individuals with elevated CAC have a 9–16 fold higher risk of CHD events compared to those with CAC = 0. A growing body of evidence shows that at least two-thirds of events are concentrated among the one fourth of the population with CAC≥100, a predictive value not seen with any other biomarker [8,9,11,12]. Recent prospective studies show that the use of CAC is associated with a net reclassification improvement for one-quarter of the whole population, and notably, in half of patients at intermediate-risk [13,14]. Although there is strong evidence for the ability of CAC to appropriately risk stratify patients, consensus on the cost-effectiveness of testing for CAC, relative to other risk assessment and treatment strategies, is not clearly established. In this study, we test the hypothesis that incorporating the results of a one-time CAC study among asymptomatic patients with intermediate-risk scores is a cost-effective means of primary CHD and CVD prevention, compared to (1) the treatment of all intermediate-risk patients, and (2) treatment based on Adult Treatment Panel III guidelines (ATP III; the current guideline at the time of this study was planned) [15]. This hypothesis is based on evidence that CAC testing improves the allocation of treatment to patients at the greatest absolute risk for CHD and CVD events, while avoiding pharmacotherapy in low-risk patients. Tailoring the use of statins to high-risk patients may be beneficial, given the potential for adverse side effects of from statin use [15]. Moreover, some patients may prefer to avoid medication, and instead use an alternative lifestyle-focused strategy for primary prevention, and therefore will experience disutility from long-term statin use [16].

## Methods

### Patient Population

We simulated an intermediate-risk subpopulation from the Multi-Ethnic Study of Atherosclerosis (MESA) (ClinicalTrials.gov registration number: NCT00005487). MESA is a community-based, prospective cohort study designed to investigate the prevalence, correlates, and progression of subclinical CVD in individuals without known CVD at baseline. Further information about the MESA study methods and the baseline clinical characteristics of our study population are provided in S1 Table. For this study, we selected MESA participants with intermediate ATP III Framingham Risk Scores of 6–20%, LDL-cholesterol levels <160 mg/dL, no current use of statins at the beginning of MESA study enrollment and no prior CHD or CVD events. We excluded patients with diabetes because the ATP III guidelines viewed this as a risk factor that requires treatment. The MESA Publications and Presentations Committee reviewed and approved this study.

### Model Structure

We developed a Markov model using TreeAge Pro 2011 healthcare software (Williamstown, MA). Our model simulated the clinical and economic effects of using a one-time CAC study to guide the treatment of intermediate-risk patients, compared to an ATP III risk-stratification strategy and a treat-all scenario, in which all intermediate-risk patients are advised to use statins. The model compared outcomes within the same cohort of patients, as if they were treated on the basis of CAC, versus the ATP III and treat-all strategies. For each strategy, we simulated outcomes over both five- and ten-year time horizons. Use of a five-year horizon aligned the model with the length of statin trials and the recommendation for CAC testing in five-year intervals for asymptomatic patients [17,18]. The ten-year horizon aligned the model with the guideline-recommended ten year risk estimate, and accounted for the likely accrual of statin benefits over the longer-term.

**Fig 1:**
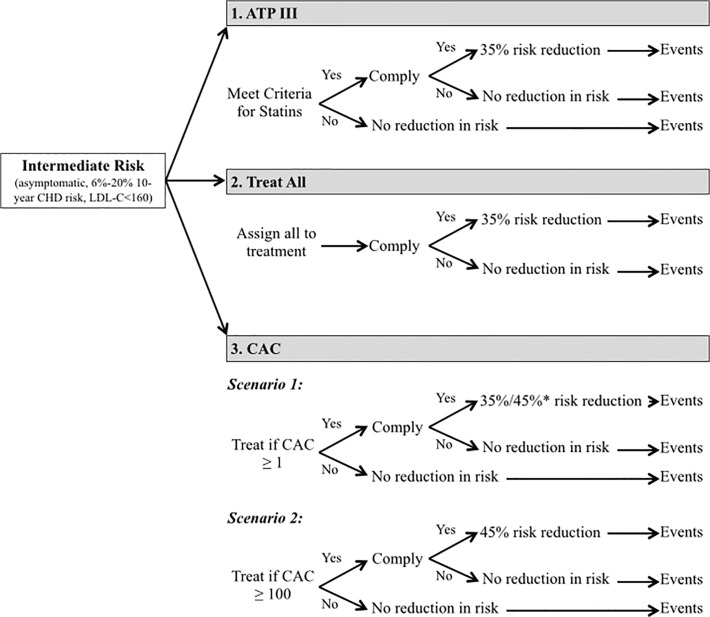
Schematic of the risk assessment and treatment strategies compared. * Patients with 1≤CAC<100 are advised to initiate standard statin therapy, which is assumed to provide a mean 35% reduction in the relative risk of CVD events. Patients with CAC≥100 are advised to begin intensive therapy, which provides a mean 45% reduction in the relative risk of CVD events.

We assessed the use of CAC to guide two treatment strategies. The first strategy recommended statin treatment in patients with CAC ≥1, and the second strategy recommended statins to patients with CAC ≥100. In the first strategy, patients with CAC scores of 1–100 were advised to begin moderate intensity statin therapy. In both CAC strategies, patients with CAC≥100 were advised to begin intensive statin therapy. Fig. 1 provides a conceptual overview of the model.

ATP III eligibility was modeled strictly following the guidelines. Eligibility was therefore based on risk factors such as LDL cholesterol levels, as well as absolute 10-year risks. When treatment was tied to CAC, CAC trumped ATP III-based treatment recommendations. For example, if a person was eligible for a statin by ATP III criteria, a statin was not prescribed in the CAC scenario if CAC = 0. In determining final ATP III treatment decisions with respect to “optional” LDL-C goals, we used a random number generator to assign a random 50% of patients who would not have been treated using standard ATP III LDL-C treated goals to statin treatment to achieve their “optional” ATP-III LDL-C goals. We then bootstrapped this randomization 100 times to determine the final ATP III statin treatment population. This randomization reflects clinical practice were only some patients with lower LDL-C levels are treated with statins to achieve even lower “optional” LDL-C levels.

We considered both CHD and CVD events as outcomes, consistent with the focus of ATP III guidelines and the new guidelines, respectively [2,3]. For the purpose of this study, CHD events consist of myocardial infarction, angina pectoris and resuscitated cardiac arrest; CVD events include all CHD events plus stroke and other cardiovascular death. We did not include intra-parenchymal hemorrhage, subarachnoid hemorrhage, or known non-atherosclerotic/non-infarct stroke. The primary outcome of interest was a first CHD or CVD event. Patients cycled through the model until experiencing an event or expiring from another cause. The model is run for five or ten cycles (depending on the time horizon), where one cycle represents one year of costs and health outcomes. Patients cycle through the model until they reach an absorbing state, which is a first CHD or CVD event. Patients who survive and accrue QALYs experience a quality-of-life decrement after the event. This decrement is assumed to diminish linearly over a two-year period following the event. All costs and outcomes were discounted annually at 3%.

We calculated effects based on two approaches of valuing health outcomes. The first valued the incremental effectiveness of CAC testing in terms of averted first CVD events, where the outcome was a binary variable (i.e., an averted event). The second valued a CVD event in terms of a reduction in utility, measured in Quality-Adjusted Life Years (QALYs). This second approach also accounted for health utility losses due to statin complications (including potential mortality from a severe adverse reaction), patients’ preferences for not taking statins, and side effects from CAC testing (mortality from radiation-induced cancer).

We calibrated the model by examining event rates by CAC group, assuming all patients were not treated (which is not a static condition of the MESA cohort), and checked that the resulting event rates equaled those observed in the MESA subsample used for this analysis.

### Data Sources

Effectiveness of treatment, cost data, and transition probabilities were obtained from published literature and the MESA study. Parameters are summarized in Table 1 along with their data sources [20–39]. We used a combination of peer-reviewed literature, the MESA database and expert opinion to specify parameters for the model, which are described below.

**Table 1:** Model Parameters.

Parameters: Probabilities and characteristics of Reclassification Groups	Base-Case Value / Mean	Distribution	Ref #
Probabilities			
Person-Year Risk of CVD Event, CAC = 0	0.00406206 (0.00413593)	Beta (approximated from the mean annual event rate)	*MESA Subsample*, *based on 5 (10)-year event rate data*
Person-Year Risk of CVD Event, 1≤CAC<100	0.01086766 (0.01115809)	Beta	*MESA Subsample*, *based on 5 (10)-year event rate data*
Person-Year Risk of CVD Event, CAC≥100	0.01920450 (0.02060006)	Beta	*MESA Subsample*, *based on 5 (10)-year event rate data*
Person-Year Risk of CHD Event, CAC = 0	0.00201915 (0.00212544)	Beta	*MESA Subsample*, *based on 5 (10)-year event rate data*
Person-Year Risk of CHD Event, 1≤CAC<100	0.00839575 (0.00756866)	Beta	*MESA Subsample*, *based on 5 (10)-year event rate data*
Person-Year Risk of CHD Event, CAC≥100	0.01676953 (0.01639898)	Beta	*MESA Subsample*, *based on 5 (10)-year event rate data*
RR of CHD/CVD Event, Normal dose of statins	.6500	Triangular (min: .55, likeliest: .65, max: .75)	Expert opinion
RR of CHD/CVD Event, High dose of statins	.5500	Triangular (min: .45, likeliest: .55, max: .65)	Expert opinion
Probability of death from CHD Event, age < 65	.10000	Beta (alpha: 17, beta:153)	Lee et al. [20]
Probability of death from CHD Event, age ≥65	.15714	Beta (alpha: 22, beta: 118)	Lee et al. [20]
Probability of death from CVD Event, age < 65	.10260	Beta (alpha: 16, beta: 140)	Lee et al. [20] and MESA Subsample Data
Probability of death from CVD Event, age ≥65	.16265	Beta (alpha: 23, beta: 118)	Lee et al. [20] and MESA Subsample Data
Probability of Mortality from Non-CHD/Non-CVD Events	US Life Table		CDC National Vital Statistics [21]
Probability of Mild Adverse Effect from Statins	0.1800	Beta (alpha: 252, beta: 1148)	Lee et al. [20]
Probability of Severe Adverse Effect from Statins	1:18000 person-years	Beta (alpha: 5.6, beta: 99994)	Lee et al. [20]
Probability of Death Given Severe Adverse Reaction	0.0900	Beta (alpha: 7.2, beta: 73)	Lee et al. [20]
Probability of Statin Adherence, No CAC Testing	0.5500	Triangular (min: .40, likeliest: .55, max: 1)	Shah [22]
Probability of Statin Adherence, CAC Testing	0.6500	Triangular (min: .50, likeliest: .65, max: 1)	Shah [22], Kalia [23], Taylor [24], Expert opinion
Lifetime Cancer Risk Due to CT-Scanning Caused Radiation Exposure	0.00002	No distribution modeled	vanKempen [25]
1-year case fatality given cancer due to radiation risk	0.6500	No distribution modeled	vanKempen [25]
Direct Medical Costs ($US 2011)			
Direct Medical Costs for Non-Fatal CHD Events	$64,400.00	Gamma (mean: $64,400; sd: $32,200)	Weighted average of condition-specific 1-year direct medical costs, where myocardial infarctions account for 49.5% of events, angina pectoris 42.6%, and Resuscitated cardiac arrests 7.9% of events; Sources: O'Sullivan, Bureau of Labor Statistics, and Bank of Canada [26–28]; and MESA Subsample Data
Direct Medical Costs for Fatal CHD Events	$49,000.00	Gamma (mean: $49,000; sd: $24,500)	Weighted average of condition-specific 3-year direct medical costs, where myocardial infarctions account for 49.5% of events, angina pectoris 42.6%, and Resuscitated cardiac arrests 7.9% of events; Sources: O'Sullivan, Bureau of Labor Statistics, and Bank of Canada [26–28]; and MESA Subsample Data
Direct Medical Costs for Non-Fatal CVD Events	$55,700.00	Gamma (mean: $55,700; sd: $27,850)	Weighted average of condition-specific 1-year direct medical costs, where stroke accounts for 25.7% of events, myocardial infarction 36.8%, angina pectoris 31.6%, and Resuscitated cardiac arrests 5.9% of events; Sources: O'Sullivan, Bureau of Labor Statistics, and Bank of Canada [26–28]; and MESA Subsample Data
Direct Medical Costs for Fatal CVD Events	$43,500.00	Gamma (mean: $43,500; sd: $21,750)	Weighted average of condition-specific 1-year direct medical costs, where stroke accounts for 25.7% of events, myocardial infarction 36.8%, angina pectoris 31.6%, and Resuscitated cardiac arrests 5.9% of events; Sources: O'Sullivan, Bureau of Labor Statistics, and Bank of Canada [26–28]; and MESA Subsample Data
Cost of CAC Testing	$75.00, $100.00, and $150.00	Triangular (min: 80% of baseline; max: 120% of baseline)	vanKempen [25] and Expert Opinion
Annual cost of statins (both intense and normal dose)	$50.00, $180.00, and $1,000.00	Triangular (min: 80% of baseline; max: 120% of baseline)	Pletcher et al. [40]
Cost of Statin Complications (mild)	$180.00	Gamma (mean: $180; sd: $30)	Extrapolated from Lee et al. [20] and vanKempen [25]
Cost of Statin Complications (severe)	$6,500.00	Gamma (mean: $6,500, sd: $3,250)	Lee et al. [20]
Total cost of follow-up for incidental non-cardiac abnormalities (incidentaolmoas)	$250.00	Gamma (mean: $250, sd: $125)	MacHaalany et al. [29]
Indirect Medical Costs			
*Average Annual Productivity Cost of a CHD Event by Age Group*:			
Age 40	$6,500.00	Gamma (mean: $6,500; sd: $3,250)	Weighted average of condition-specific productivity costs, where myocardial infarction accounts for 49.5% of events, angina pectoris 42.6%, and Resuscitated cardiac arrests account for 7.9% of events; Source: Grover [30]
Age 50	5,100.00	Gamma (mean: $3,100; sd: $1,550)	Source: Grover [30]
Age 60	1,900.00	Gamma (mean: $1,900; sd: $800)	Source: Grover [30]
Age 70	500.00	Gamma (mean: $500; sd: $250)	Source: Grover [30]
Age 80	200.00	Gamma (mean: $200; sd: $100)	Source: Grover [30]
Time Cost of CAC Testing (assumed to be 1 hour)	15.20	Gamma (alpha: 1.056 lambda: .152)	Bureau of Labor Statistics [31]
*Note*: *Linear interpolation is used to estimate the mean productivity cost of a CHD event at intermediate ages*. *The standard deviation of the gamma distribution is 50% of the mean cost*. *For CVD events (which include stroke)*, *the age-specific mean annual productivity costs were*: *$7*,*900 (age 40)*, *$6*,*200 (age 50)*, *$2*,*300 (age 60)*, *$600 (age 70)*, *and $200 (age 80)*. *These estimates were computed as a weighted average of event-specific event rates*, *where the weights reflect the proportion of CVD events occurring in the MESA subsample (see notes to direct cost estimates for CVD events)*.			
Health Utility Values			
*Age-Specific QALY Values*, *Healthy*:			
Age 50	0.8400	No distribution modeled	Lee et al. [20]
Age 60	0.8200	No distribution modeled	Lee et al. [20]
Age 70	0.7900	No distribution modeled	Lee et al. [20]
Age 80	0.7400	No distribution modeled	Lee et al. [20]
Age 90	0.6800	No distribution modeled	Lee et al. [20]
*Note*: *Linear interpolation was used to estimate age-specific healthy QALYs at intermediate ages*.			
*Factors by which age-specific QALYs were multiplied to reflect the occurrence of adverse events or the general disutility of taking a statin*:			
General Disutility from Taking Statin	0.99616	Triangular (min: 0.99232, likeliest: 0.99616, max: 1.000)	Pletcher et al. [40]
Mild Statin Complications (Annual health utility loss)	0.9941	Triangular (min: 0.9986, likeliest: 0.9941, max: 0.9890)	Lee et al. [20]
Severe Statin Complications (Annual health utility loss)	0.9553	Triangular (min: 0.9808, likeliest: 0.9553, max: 0.9233)	Lee et al. [20]
Nonfatal CHD Event (Annual health utility loss)	0.8351	Beta (alpha: 102, beta: 20)	Weighted average of CHD condition-specific health utility losses, obtained from Lee et al. [20]. Weights based on events in the MESA subsample.
Nonfatal CVD Event (Annual health utility loss)	0.8272	Beta (alpha: 180, beta: 38)	Weighted average of CVD condition-specific health utility losses, obtained from Lee et al. [20]. Weights based on events in the MESA subsample.
No Statin Complications	1.0000	N/A	-
Death	0.0000	N/A	-

### MESA Study and Event Rates

The proportion of individuals whose risk was reclassified following a CAC test is shown in Table 2. If patients with CAC ≥1 were advised to use statins, CAC-based treatment increased the proportion of the intermediate-risk population that was statin-eligible by 20%. If only patients with CAC ≥100 were advised to use statins, then 10.3% fewer intermediate-risk patients were eligible for a statin.

**Table 2:** Re-Classification of ATP III Assessed Statin Eligibility by CAC.

	Risk as Assessed by CAC (Assumed Gold Standard)				
Risks as Assessed by ATP III		Highest Risk	At Risk	Not at Risk	Total
		CAC ≥ 100	1 ≤ CAC < 100	CAC = 0	(All CAC Groups)
	**Statin Eligible by ATP III:**				
	N	193	196	226	615
	% of Total Population	11.9%	12.1%	14.0%	
	**Non Statin Eligible by ATP III:**				
	N	256	294	454	1,004
	% of Total Population	15.8%	18.2%	28.0%	
	**Total:**				
	Total (All ATP III Groups):	449	490	680	1,619
**Summary Statistics**					
**Statin Treatment Advised for CAC ≥ 1**					
% of Population Reclassified as At Risk or Highest Risk via CAC:					34.0%
% of Population Reclassified as Not At Risk or Highest Risk via CAC:					14.0%
Net % Reclassification to At Risk or Highest Risk via CAC:					20.0%
**Statin Treatment Advised for CAC ≥ 100**					
% of Population Reclassified as Highest Risk via CAC:					15.8%
% of Population Reclassified as Not Highest Risk via CAC:					26.1%
Net % Reclassification to At Highest Risk via CAC:					−10.3%

Note: Authors’ calculations from the Multi-Ethnic Study of Atherosclerosis.

We assumed that CHD and CVD event rates varied by CAC score. Event rates in our model reflected mean annual rates of first events in three CAC strata (CAC = 0, 1≤CAC<100, CAC≥100), and were based on the subpopulation from MESA chosen for this analysis. The five-year time horizon models were based on average annual event rates over five years; the ten-year models used average annual rates over ten years.

### Effectiveness and Treatment Adherence

We modeled the benefit of statins based on the results of recent meta-analyses of randomized controlled trials on statin efficacy [18,36]. Individuals in the treat-all scenario were assigned to moderate statin therapy, which was modeled as providing a mean 35% relative reduction in risk. Individuals in the ATP III arm of the model who qualified for statin treatment were also recommended to receive moderate intensity statins. In the CAC component of the model, patients with CAC ≥100 were modeled as initiating intensive statin treatment and receiving a 45% relative risk reduction; those with 1≤CAC<100 received moderate intensity statin therapy. No statin dose adjustments or tapering of statin efficacy were assumed over the duration of the model [19].

We assumed a 55% rate of statin adherence when treatment was guided by ATP III [22]. Research indicates that patients who visualize moderate calcium deposits in the coronary artery have a significantly higher rate of adherence [22–24]. Therefore, the mean rate of adherence was assumed to increase by approximately 10% among patients undergoing CAC scanning.

The case-fatality rate from a first CHD event was assumed to be 10.0% for adults under age 65, and 10.3% for non-elderly adults experiencing CVD events. We assumed a discrete increase in the risk of CHD-attributable mortality in individuals age 65 and above [20]. In addition to mortality from CHD events, we modeled non-CHD death using age-specific mortality rates from the CDC’s US life table [21].

### Adverse Outcomes

We modeled outcomes as both averted CHD or CVD events, valued as counts, and in QALYs. QALYs captured losses in health due to CHD or CVD events, as well as side effects from statins and the disutility of ongoing statin use. By disutility, we mean a patient’s inherent desire to avoid use of a medication that may not improve health. QALYs lost from any of these outcomes were modeled as proportionate reductions in an individual’s age-specific utility associated with full health, and reflected both the severity and duration of the outcome (in relation to a year). We modeled mild and severe statin complications, using utility decrements of 2 days and 2 weeks of lost healthy life, respectively [20]. Lastly, CAC testing entails exposure to a modest dose of ionizing radiation (in general, approximately 1mSv) [32,33]. We assumed a modest incremental increase in lifetime cancer risk due to the CT scan used to test for CAC. We allocated the portion of this cumulative risk that accrues over the time horizon of the model using a linear approximation to an exponential model [25].

### Incidental Findings

CAC testing may uncover incidental non-cardiac findings that warrant follow-up examinations. Studies have found that these incidental findings are detected and reviewed through follow-up tests in 4%-8% of patients. Although incidental findings could reveal early-stage cancers, studies have found that very few non-calcified lung nodules ultimately become cancerous. Therefore, in a sensitivity analysis, we included a cost penalty for re-scanning 8% of patients in the CAC testing arm, which is based on a study of re-scanning rates in patients tested for CAC. In this sensitivity analysis, we conservatively assumed no future health or cost benefits from the follow-up scans [29,34,35].

### Costs

Cost data were obtained from literature searches and expert consultation. In selecting cost estimates, we assessed the published literature on the basis of: (1) comparability of the study’s population to ours, (2) rigor in methods used to determine incremental costs attributable to an event, and (3) the year of the study. We ultimately selected a 2007 study using Medicare Advantage data to estimate the direct medical costs attributable to CVD events [26], and a 2002 Canadian study to determine age-specific productivity losses due to events [30]. We estimated direct medical costs and productivity costs of a CHD or CVD event as a weighted average of costs for specific events, using the relative frequencies of event types (e.g., angina pectoris or myocardial infarction) in the MESA sample as weights. Costs were converted to same-year US dollars, where applicable, and then inflated to 2011 dollars using the CPI for Medical Care [27,28]. We assumed the productivity cost of CAC testing to be 1 hour, valued at the US median hourly wage rate for workers over age 55 (calculated as the 2011 median weekly wage for workers ≥ age 55, divided by average 2011 weekly hours for workers ≥ age 55) [31].

Base-case simulations included the direct costs of CAC testing, statins, and CHD or CVD events, but excluded productivity costs and the cost of re-scanning patients with incidental findings. The base-case analyses assumed a mean direct cost of $100 for a CAC test and $180 annually for the use statins (both entered into the model as triangular distributions, and varied by +/− 20%). Costs reflect anticipated payments to providers, instead of initial charges, which may be higher.

The main results are reported using all base-case parameter assumptions, separately for CHD and CVD events, for scenarios where patients with CAC≥1 are treated, and again where only patients with CAC≥100 are treated. We report incremental costs per averted event and per QALY for these simulations.

### Sensitivity Analyses

Broadly, we conducted two types of sensitivity analyses. The first was a probabilistic sensitivity analysis that simulated uncertainty in model parameters. Table 1 specifies distributions for the main transition probabilities, health utilities, and costs. We drew values from each distribution for 2,000 hypothetical patients, whose outcomes we then simulated in the model, and repeated this process over 1,000 simulations. We evaluated the mean and, for certain models, the distribution of costs and effects across the 1,000 simulations, for each strategy. We compared incremental costs and incremental effects for the three possible pairwise comparisons of interventions. A ratio of mean incremental costs and incremental effects for a particular pair of strategies is reported as an incremental cost-effectiveness ratio (ICER).

For the other type of sensitivity analyses, we considered changes to assumptions about specific parameters of the model, as follows. First, we considered the effect of shifting the mean cost of a CAC test to $75, $150, and $250. Second, we changed the mean annual cost of statins to $50 and $1,000. The higher end of the statin cost range may account for costs of follow-up physician visits and laboratory tests associated with statin use, and the prescription of brand name statins. Third, we included productivity costs for CAC testing and events, as well as the downstream cost of re-examining patients with incidental non-cardiac findings.

Lastly, we considered the effect of changing CVD and CHD event rates to more closely resemble those in the general US adult population. In MESA and other prospective studies, event rates tend to be lower than in the general population. This difference may reflect the fact that a prerequisite for participation in MESA is survival from mortality risk factors, including CVD, as well as the fact that some MESA subjects initiated statin use after enrolling in the study. A comparison of event rates in MESA to nationally representative data suggests that MESA event rates are one and one-half to two times lower than among similarly aged adults in the general population [1]. Therefore, we compared our base-case set of results with models that assumed annual events rates two times higher than the base-case set of MESA-derived rates. The probabilistic sensitivity analysis and sensitivity analyses on the cost parameters were repeated in the 2x MESA rates simulations.

## Results

In our base-case simulations, we project that, compared to an ATP III-based treatment approach, testing all intermediate risk patients for CAC and treating those with CAC ≥1 averts an average of 5.1 additional first CHD events and 5.8 additional first CVD events per 1,000 patients over five years. Treating all intermediate risk patients averts an additional 3.9 CHD events and an additional 5.1 CVD events per 1,000 patients over five years, in comparison to ATP III (Table 3). Treating patients on the basis of CAC is more effective, in terms of averted events, than treating all intermediate risk patients, because CAC enables providers to identify candidates for intensive statin therapy, and because patients are assumed to be more adherent to statins in the CAC strategies. The results are similar, although larger in magnitude, over a ten-year outlook.

**Table 3:** Averted CHD and CVD Events Per 1,000 Persons, Base-Case MESA Event Rates.

	ATP III (Events)	Treat All (Δ Events, Compared to ATP III)	CAC* (Δ Events, Compared to ATP III)
5 Years			
CHD Events	31.7	27.9 Δ = −3.9)	26.7 Δ = −5.1)
CVD Events	40.8	35.7 Δ = −5.1)	35.0 Δ = −5.8)
10 Years			
CHD Events	52.3	46.1 Δ = −6.2)	44.5 Δ = −7.9)
CVD Events	72.7	64.0 Δ = −8.6)	62.9 Δ = −9.8)

Note: Simulated events per 1,000 persons, by risk assessment and treatment strategy. The results displayed in this table value outcomes in terms of averted events, but *not* QALYs. Results reflect all base-case model assumptions and 1x MESA event rates.

* Column displays results for the scenario where patients with CAC≥1 are advised to initiate statins (intensive therapy for CAC≥100, and standard therapy for 1≤CAC<100).

Table 4 shows the risk assessment and treatment strategies that would be selected under different assumptions about costs and the valuation of outcomes, using base-case MESA event rates. The mean costs and effects calculated under each scenario are provided in S2 Table. (Corresponding scenarios are identified by the row numbers of Table 4 and S2 Table). Screening all intermediate-risk patients and treating those with CAC ≥1 is generally the least costly and most effective strategy, compared to treat all and ATP III, if the mean cost of a CAC test is $150 or below. The break-even point, at which CAC becomes cost-ineffective compared to ATP III, is approximately $235/test. Limiting statin therapy to patients with CAC ≥100 averts fewer events than a treat all scenario, such that treating all patients is preferred to both the CAC and ATP III strategies, if outcomes are valued only as averted events. If annual statin costs average $1,000, treating all patients becomes too costly to implement at a willingness-to-pay threshold of $100,000/averted event or lower. In this scenario, ATP III is favored over treating patients with CAC ≥1 and treating all intermediate-risk patients.

**Table 4:** Results Using Base-Case MESA Event Rates.

Scenario	Mean CAC Scan Cost ($)	Mean Annual Statin Cost ($)	Time Horizon (years)	Treat CAC≥100	Valuation of Outcomes	Decision
CHD Events						
1	100	180	5	No	Events	CAC Dominates Both
2	100	180	5	No	QALYs	CAC Dominates Both
3	100	180	5	Yes	Events	Treat All Dominates ATP III
4	100	180	5	Yes	QALYs	CAC Dominates Both
5	100	180	10	No	Events	CAC Dominates Both
6	100	180	10	No	QALYs	CAC Dominates Both
7	100	180	10	Yes	Events	Treat All Cost-Effective; ICER = $4,373
8	100	180	10	Yes	QALYs	CAC Dominates
Sensitivity Analyses on Cost Parameters						
9	100	50	5	No	Events	CAC Dominates Both
10	100	50	5	No	QALYs	CAC Dominates Both
11	100	1,000	5	No	QALYs	ATP III (Status Quo)
12	100	1,000	5	Yes	QALYs	CAC Dominates Both
13	75	180	5	No	QALYs	CAC Dominates Both
14	75	180	5	Yes	QALYs	CAC Dominates Both
15	150	180	5	No	QALYs	CAC Dominates Both
16	150	180	5	Yes	QALYs	CAC Dominates Both
17	250	180	5	No	QALYs	ATP III (Status Quo)
18	Base-Case Assumptions + Indirect Costs & Incidentalomas (QALYs)					CAC Dominates Both
CVD Events						
19	100	180	5	No	Events	CAC Dominates Both
20	100	180	5	No	QALYs	CAC Dominates Both
21	100	180	5	Yes	Events	Treat All Dominates ATP III
22	100	180	5	Yes	QALYs	CAC Dominates Both
23	100	180	10	No	Events	CAC Dominates Both
24	100	180	10	No	QALYs	CAC Dominates Both
25	100	180	10	Yes	Events	Treat All Dominates ATP III
26	100	180	10	Yes	QALYs	CAC Dominates Both
Sensitivity Analyses on Cost Parameters						
27	100	50	5	No	Events	CAC Dominates ATP III
28	100	50	5	No	QALYs	CAC Dominates ATP III
29	100	1,000	5	No	QALYs	ATP III (Status Quo)
30	100	1,000	5	Yes	QALYs	CAC Dominates Both
31	75	180	5	No	QALYs	CAC Dominates Both
32	75	180	5	Yes	QALYs	CAC Dominates Both
33	150	180	5	No	QALYs	CAC Dominates Both
34	150	180	5	Yes	QALYs	CAC Dominates ATP III
35	250	180	5	No	QALYs	ATP III (Status Quo)
36	Base-Case Assumptions + Indirect Costs & Incidentalomas (QALYs)					CAC Dominates Both

Note: A risk assessment and treatment strategy is said to dominate if it is less costly and more effective than both of the alternative strategies to which it is compared. Otherwise, the favored strategy may be incrementally more costly and more effective than ATP III, which was the standard of risk assessment when this study was conducted. If the incremental cost per unit of effect is less than or equal to $50,000, but positive, the alternative intervention is assumed to be favored, and an incremental cost-effectiveness ratio (ICER) is reported. If the ICER exceeds $50,000, ATP III is preferred. Mean costs and effects for each strategy, which are the basis for the decisions summarized in the table, are presented in S2 Table. Scenarios are identified by the scenario number on each row of the table.

When outcomes are valued in QALYs, we project that a CAC-based treatment strategy is consistently preferred to a treat-all strategy. Although more CHD and CVD events occur when statins are recommended only in the highest-risk patients (i.e., those with CAC ≥100), treating patients with CAC≥100 produces a greater net gain in QALYs than the treat-all strategy, because statin use is limited to individuals who are likely to experience the greatest benefit from therapy (see Scenario 4). Statin use, and any accompanying negative side effects, is avoided in persons with CAC below 100 in this scenario. These findings are robust to the inclusion of indirect costs and the cost of re-examining patients with incidentalomas. Table 5, which shows the results of simulations that are based on 2x MESA event rates, also reaches similar conclusions. Corresponding mean costs and effects are summarized in S3 Table.

**Table 5:** Sensitivity Analysis on Event Rate Parameters—2x MESA Event Rates.

Scenario	Mean CAC Scan Cost ($)	Mean Annual Statin Cost ($)	Time Horizon (years)	Treat CAC≥100	Valuation of Outcomes	Decision
CHD Events						
37	100	180	5	No	Events	CAC Dominates Both
38	100	180	5	No	QALYs	CAC Dominates Both
39	100	180	5	Yes	Events	Treat All Dominates ATP III
40	100	180	5	Yes	QALYs	CAC Dominates Both
41	100	180	10	No	Events	CAC Dominates Both
42	100	180	10	No	QALYs	CAC Dominates Both
43	100	180	10	Yes	Events	Treat All Dominates ATP III
44	100	180	10	Yes	QALYs	CAC Dominates Both
CVD Events						
45	100	180	5	No	Events	CAC Dominates Both
46	100	180	5	No	QALYs	CAC Dominates Both
47	100	180	5	Yes	Events	Treat All Dominates ATP III
48	100	180	5	Yes	QALYs	CAC Dominates ATP III
49	100	180	10	No	Events	CAC Dominates Both
50	100	180	10	No	QALYs	CAC Dominates Both
51	100	180	10	Yes	Events	Treat All Dominates Both
52	100	180	10	Yes	QALYs	CAC Dominates ATP III

Note: A risk assessment and treatment strategy is said to dominate if it is less costly and more effective than both of the alternative strategies to which it is compared. Otherwise, the favored strategy may be incrementally more costly and more effective than ATP III, which was the standard of risk assessment when this study was conducted. If the incremental cost per unit of effect is less than or equal to $50,000, the alternative intervention is assumed to be favored, and an incremental cost-effectiveness ratio (ICER) is reported. If the ICER exceeds $50,000, but is positive, then ATP III is preferred. Mean costs and effects for each scenario, which are the basis for the decisions summarized in the table, are presented in S3 Table. Scenarios are identified by the scenario number on each row of the table.

**Fig 2:**
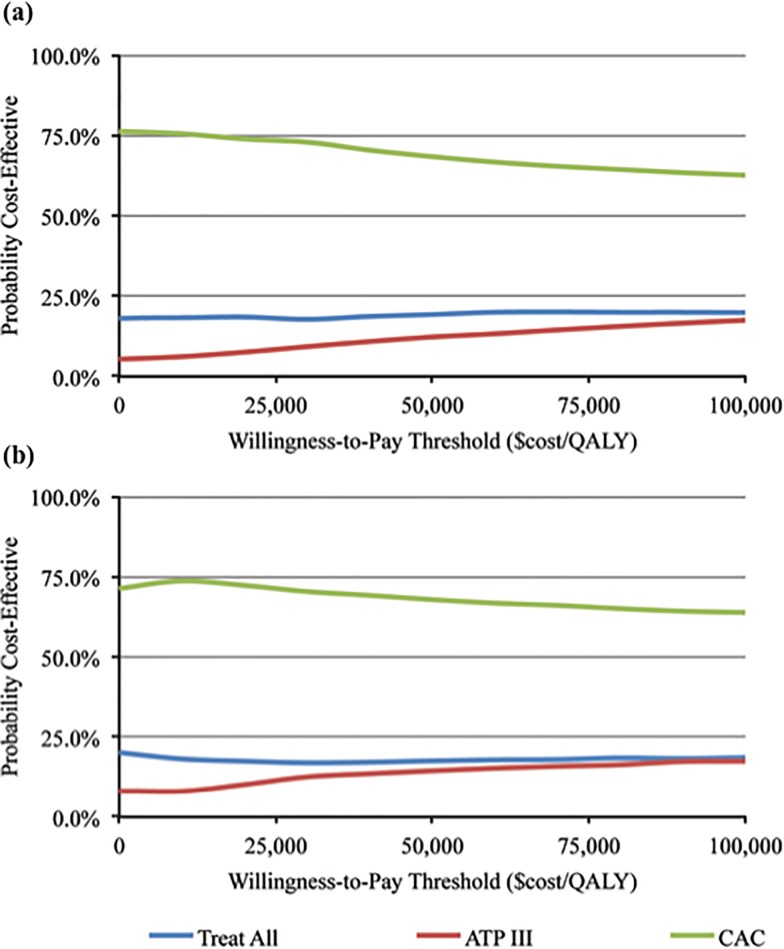
Cost-Effectiveness Acceptability Curves. Panel (a): 10-Year CVD Events, Treat CAC ≥ 1. Panel (b): 10-Year CVD Events, Treat CAC ≥ 100. Note: The cost-effectiveness acceptability curves show the proportion of simulations (vertical axis) that are cost-effective at a given willingness-to-pay threshold (horizontal axis). A mean CAC scanning cost of $100 and a mean statin cost of $180 is assumed in both plots (indirect costs and costs associated with incidentalomas are not included). The vertical intercept of each cost-effectiveness acceptability curve includes simulations that are cost saving and which result in a loss of fewer QALYs compared to the alternative scenarios. The intercept can be interpreted as the probability that a strategy would be accepted at a willingness-to-pay threshold of $0/QALY. For example, approximately 75% of simulations in both CAC strategies would be accepted at the $0/QALY threshold.

Fig. 2 plots the cost-effectiveness acceptability curves for each risk assessment and treatment strategy, for the prevention of CVD events over a ten-year horizon, when outcomes are valued as QALYs. The figure summarizes the distribution of the simulated results, based on the proportion of simulations that are cost-effective (measured on the vertical axis) at different willingness-to-pay thresholds (horizontal axis). The intercept of each curve with the vertical axis represents the proportion of simulations for a given strategy that would be accepted at a willingness-to-pay threshold of $0/QALY; the intercept includes simulations for which a strategy is both cost saving and more effective than the two other alternatives. The figure demonstrates that approximately 75% of the CAC simulations are cost-effective at the $0/QALY threshold, compared to the treat-all and ATP III strategies. CAC remains favored in a majority of simulations at positive willingness-to-pay thresholds.

## Discussion

We investigated the cost-effectiveness of CAC testing in intermediate-risk individuals, to predict CHD and CVD risks, and to guide statin allocation. Using a Markov simulation model, we compared a CAC strategy to treat-all and ATP III strategies for screening and treatment. We found that CAC testing is generally less costly and more effective than these alternative strategies, particularly when we account for the effects of adverse statin reactions and the disutility of taking a statin. A CAC-based strategy permits identification of appropriate candidates for statin therapy, enables clinicians to adjust the intensity of the therapy to patients’ risk, and avoids pharmacological intervention in a large group of truly lower risk patients.

CAC is known to be strongly predictive of absolute risks for CHD and CVD. When evaluating the effectiveness of screening and treatment strategies on the basis of averted events, treating patients with any CAC (i.e., CAC ≥1) was preferred to the alternative strategies we considered. Even at a higher CAC cost of $150, which is about double the available cost of a CAC study in certain US cities, such as Baltimore and Miami, CAC testing remained cost-effective at 1x and 2x MESA event rates. Limiting statin treatment to patients with CAC ≥100 prevented fewer CHD and CVD events, but reduced the number of patients who might disfavor taking a statin, and the occurrence of adverse statin side effects. Consequently, CAC screening and treatment of just the highest-risk patients (CAC ≥100) was favored when outcomes were valued in QALYs. This result is consistent with a prior MESA study, which suggested that accounting for the adverse effects of statin use could make a treat-all strategy less attractive than more selective treatment [40]. The American College of Cardiology and American Heart Association (ACC/AHA) recently released new cholesterol [7] and risk assessment guidelines [2], which have important implications for this analysis. In a primary prevention patient considered for statin therapy, the 2013 ACC/AHA guidelines recommend that a risk discussion occur if the low-density lipoprotein cholesterol level is 70–189 mg/dL and if the estimated 10-year atherosclerotic cardiovascular disease risk is 5% or higher. The vast majority of intermediate risk patients included in our analysis have a 10-year risk of 5% or higher under the new guidelines. Our analysis suggests that performing a CAC study could add constructively to discussions about risk and appropriate treatment. While the risk discussion is also likely to incorporate factors that we do no model in this study, our results suggest that CAC may be helpful in adjudicating treatment decisions, particularly when patients or providers are concerned about the disadvantages of statin use, or the appropriate intensity of therapy if treatment is initiated [5–7]. This analysis extends prior contributions to the cost-effectiveness literature on statins and CAC. Lazar et al. concluded that a broad treatment expansion using low-cost statins could avoid 6.3% of all CHD deaths in the United States at a favorable cost-effectiveness ratio [39]. An analysis by Sniderman et al. also compared CAC testing to a strategy that called for broader statin therapy for primary prevention [10]. Their analyses indicated that the number needed to treat to reduce CHD events by 23% was 70 for a treat-all with moderate intensity statin scenario, and 43 for CAC-guided scenario. While highlighting that CAC testing permits more efficient allocation of pharmacotherapy, by requiring statin use in fewer patients to reduce an equal number of events, the conclusions about cost-effectiveness were limited by the study’s assumptions. The analysis seemed to favor broad statin treatment, but assumed a high CAC testing cost of $340, an unrealistic medication adherence rate of 100%, and no negative statin side effects or disutility from taking a medication.

A cost-effectiveness analysis by van Kempen et al. from the Rotterdam study found CAC testing to be cost-effective for men (ICER = $48,000/QALY), but also concluded that broader statin therapy was less costly per QALY than CAC testing [25]. Several important factors may account for the difference between these conclusions and ours. First, the population in the Rotterdam study is older than in MESA, with a mean age of 70 among men and 74 among women. Second, the modeling used a patient’s remaining lifetime as the analytic horizon, extrapolating CHD incidence, as well as statin effectiveness, beyond available data to make projections over this long analytic horizon. Third, the van Kempen study modeled the synergistic pharmacologic effect of statins, anti-hypertensives and in certain instances aspirin for CHD primary prevention, while we focused on the incremental benefit of statin use alone. Fourth, we model the plausible assumption that clinicians will provide high-dose statins to patients in whom very high CAC is detected, resulting in more effective statin treatment for these patients.

Pletcher and colleagues recently published a cost-effectiveness of CAC to guide statin therapy in intermediate risk persons from MESA [40]. Using a base-case 10-year horizon, and 55-year-old men and women, it was concluded that CAC could be cost-effective only in the setting of high cost statin therapy or significant negative effects on quality of life. A CAC treatment threshold of 0 was suggested. Our analysis clarifies the impact of using different intensities of statin therapy their related risk reductions based on the magnitude of CAC elevation. In addition, our study adds greater clarity about thresholds for the cost of statins and CAC tests that may make a strategy cost-prohibitive.

Our results conservatively represent the benefits of CAC testing over a period of reliable data, and in a population that is more ethnically diverse, and representative of the US, than prior studies. We also consider a variety of patient characteristics and policy parameters under which testing is most likely to be cost-effective. While we consider the cost-effectiveness of testing patients for CAC based on standard willingness-to-pay thresholds, we also consider more general thresholds (see Fig. 2). This helps to account for the fact clinicians may judge the long-term benefits of statin therapy to further offset the upfront cost of CAC scanning, and addresses a longstanding debate over the appropriateness of the $50,000 threshold in the cost-effectiveness literature [41,42]. *Limitations*

Our model makes assumptions about clinical outcomes, their corresponding costs and effects on patients’ quality of life, and potential correlations between parameters. We focus on the primary prevention of CHD and CVD effects through statin use. Although modeling CVD was important in light of the new ACC guidelines, we acknowledge that there is lack of clarity with about which proportion are non-atherosclerotic, and therefore less responsive to statin therapy.

We conducted probabilistic sensitivity analyses for the main cost, outcome and probability parameters, but the distributions and ranges tested may not represent the range of possible values for all patient populations. For instance, we varied the relative risk reduction of attributable to moderate-intensity statin statins from 25–45%, and the risk-reduction from high-dose statins from 35%-55%, based on overall estimates from meta-analyses of randomized controlled trials. However, not vary these proportional benefits in relation to specific clinical characteristics, such as baseline LDL cholesterol levels [45]. In addition, we model the cost of CHD and EVD events based on data from Medicare Advantage plans. Costs in Medicare Advantage populations have been reported to be lower than in the Medicare fee-for-service population, likely due to favorable risk selection into managed care plans [43]. Moreover, the estimates in our modeling were driven from multiple previous publications and it is important to consider that estimates can be variable from study to study based on population characteristics and inclusion criteria. We did not take into account any potential synergistic benefit with anti-hypertensive regimens, and more controversially, with aspirin in certain patients, nor did we model other potential non-CVD benefits of statin use, such as nephroprotection or prevention of dementia [44]. In addition, a sizable percentage of patients in the MESA population were potentially started on statin treatment subsequent to the study enrollment, and participants may also be healthier than similarly aged adults in the US. We attempted to account for these factors by re-running our simulations with elevated event rates.

Our model does not simulate a cohort of patients for the remainder of their lives. As such, we did not examine the downstream impact of preventing a first CHD or CVD event, and did not test the implications of treating patients with statins for the remainder of their lives. These long-term consequences are difficult to estimate with existing data. We held to five- and ten-year time horizons. The 5-year horizon is most conservative and matches the typical length of randomized controlled trials testing the efficacy of statins. Extending the time horizon beyond 5 years takes the analysis outside of the available randomized trial data, and requires us to make an assumption on the long-term effectiveness of statins. However, this analysis allows us to consider the downstream benefits of statin use. Finally, we do not model the interaction of statins with aspirin use and other primary prevention strategies.

## Conclusion

The intended use population for the estimates from our study is one that is intermediate risk, based on traditional risk factors. Using conservative parameters in a simulation model, we find that CAC testing in intermediate-risk patients is likely to be cost-saving and more effective than both treat all and ATP III-based strategies, over five- and ten-year time horizons. CAC may enable providers to better refine risk based treatment decisions in intermediate-risk patients, and enhance providers’ ability to implement CVD prevention guidelines.

## Supporting Information

S1 Methods Background on the Multi-Ethnic Study of Atherosclerosis. (DOCX)

S1 Table Baseline Characteristics of Study Population. Note: Data are presented as mean +/− standard deviation, median (25th, 75th percentile), or No. (%). CHD = coronary heart disease; HDL = high-density lipoprotein; LDL = low-density lipoprotein; CAC = coronary artery calcium score. Source: Authors’ calculations from the Multi-Ethnic Study of Atherosclerosis.
(DOCX)

S2 Table Results Using Base-Case MESA Event Rates. Note: The results presented are mean costs and effects calculated over 1,000 simulations. Negative numbers in the mean effects columns are counts of events or losses of QALYs (depending on the valuation of outcomes). Italicized lines indicate that only patients with CAC≥100 are treated in the CAC strategy. ICER = Incremental Cost-Effectiveness Ratio. The model scenario numbers correspond to the scenarios presented in Table 4.
(DOCX)

S3 Table Sensitivity Analysis on Event Rate Parameters—2x MESA Event Rates. Note: The results presented are mean costs and effects calculated over 1,000 simulations. Negative numbers in the mean effects columns are counts of events or losses of QALYs (depending on the valuation of outcomes). Italicized lines indicate that only patients with CAC≥100 are treated in the CAC strategy. The model scenario numbers correspond to the scenarios presented in Table 5.
(DOCX)
