# Supplementary material for: Cost-Effectiveness of Coronary Artery Calcium Testing for Coronary Heart and Cardiovascular Disease Risk Prediction to Guide Statin Allocation: The Multi-Ethnic Study of Atherosclerosis (MESA)
Source: PLoS One. 2015 Mar 18;10(3):e0116377. doi: 10.1371/journal.pone.0116377 (PMC4364761; doi:10.1371/journal.pone.0116377)
Supplement: S1 Methods — (DOCX) [file pone.0116377.s001.docx]

S1 Methods: Background on the Multi-Ethnic Study of Atherosclerosis

The Multi-Ethnic Study of Atherosclerosis (MESA) includes 6814 women and men aged 45 to 84 years without known CVD, recruited from 6 US communities (Baltimore, Maryland; Chicago, Illinois; Forsyth County, North Carolina; Los Angeles County, California; northern New York City, New York; and St. Paul, Minnesota). MESA is racially and ethnically representative. Baseline sociodemographic and clinical characteristics of the MESA subpopulation included in this study are shown in Table S2.

Diabetes was defined as self-reported history of diabetes mellitus, diabetes medication use, or a fasting glucose level of 126 mg/dL or greater.A Demographics, medical history, anthropometric and laboratory data for the present study were taken from the first examination (July 2000 to August 2002). Current smoking was defined as having smoked a cigarette in the last 30 days. Use of antihypertensive and other medications was based on review of prescribed medication containers. Resting blood pressure was measured 3 times in the seated position and the average of the second and third readings was recorded. Hypertension was defined as a systolic blood pressure of at least 140 mm Hg, diastolic blood pressure of at least 90 mm Hg, or use of medication prescribed for hypertension.

Body mass index was calculated as weight in kilograms divided by height in meters squared. Total cholesterol and high-density lipoprotein (HDL) cholesterol were measured from blood samples obtained after a 12-hour fast. LDL cholesterol was estimated using the Friedewald equation. Family history of CHD was obtained by asking participants whether any member in their immediate family (parents, siblings, and children) experienced fatal or nonfatal myocardial infarction. The MESA study was approved by the institutional review boards of each study site and written informed consent was obtained from all participants.

*Measurement of the CAC Score*

CT scanning and interpretation methods in MESA were reported by Carr et al. Scanning centers assessed CAC by chest computed tomography (CT) with either a cardiac-gated electron-beam CT scanner (Chicago, Los Angeles County, and New York City field centers) or a multi-detector CT system (Baltimore, Forsyth County, and St Paul field centers). Certified technologists scanned all participants twice over phantoms of known physical calcium concentration. A radiologist or cardiologist read all CT scans at a central reading center (Los Angeles Biomedical Research Institute at Harbor–UCLA, Torrance, California). Intra-observer and inter-observer agreements were excellent (κ=0.93 and κ=0.90). We used the mean Agatston score for the 2 scans in all analyses.

*Follow-up*

New occurrences of CHD were recorded over a mean follow-up of 7.1 (± 1) years. At intervals of 9 to 12 months, an interviewer contacted each participant or family member by telephone to inquire about interim hospital admissions, outpatient diagnoses of CHD and CVD, and deaths. To verify self-reported diagnoses, MESA obtained medical records for approximately 98% of hospital events and 95% of outpatient diagnoses. For out of hospital cardiovascular deaths, next of kin were interviewed and copies of death certificates were requested.
