# Supplementary material for: Cost-Effectiveness of Coronary Artery Calcium Testing for Coronary Heart and Cardiovascular Disease Risk Prediction to Guide Statin Allocation: The Multi-Ethnic Study of Atherosclerosis (MESA)
Source: PLoS One. 2015 Mar 18;10(3):e0116377. doi: 10.1371/journal.pone.0116377 (PMC4364761; doi:10.1371/journal.pone.0116377)
Supplement: S1 Table — Note: Data are presented as mean +/− standard deviation, median (25th, 75th percentile), or No. (%). CHD = coronary heart disease; HDL = high-density lipoprotein; LDL = low-density lipoprotein; CAC = coronary artery calcium score. Source: Authors’ calculations from the Multi-Ethnic Study of Atherosclerosis. (DOCX) [file pone.0116377.s002.docx]

S1 Table: Baseline Characteristics of Study Population

|  | N=1619 |
| --- | --- |
| Age, years | 65.2 +/- 9.5 |
| Sex |  |
| Women | 434 (26.8%) |
| Men | 1,185 (73.2%) |
| Race/ethnicity |  |
| White | 652 (40.3%) |
| Chinese | 204 (12.6%) |
| African American | 426 (26.3%) |
| Hispanic | 337 (20.8%) |
| Body mass index, kg/m^2^ | 27.5 +/- 4.7 |
| Smoking status |  |
| Never | 746 (46.1%) |
| Former | 647 (40.0%) |
| Current | 226(13.9%) |
| Completed high school | 1312 (80.1%) |
| Family history of CHD | 634, 39.35% |
| Systolic blood pressure, mm Hg | 129 +/- 21 |
| Diastolic blood pressure, mm Hg | 74 +/- 10 |
| Anti-hypertensive medication | 567 (35.0%) |
| Total cholesterol, mg/dL | 189 +/- 28 |
| HDL cholesterol, mg/dL | 49 +/-14 |
| Triglycerides, mg/dL | 106 (74, 156) |
| LDL cholesterol (Friedewald) | 115 +/- 25 |
| CAC score |  |
| Mean +/- SD | 155 +/- 404 |
| Median (25^th^, 75^th^) | 11 (0, 122) |

Note: Data are presented as mean +/- standard deviation, median (25^th^, 75^th^ percentile), or No. (%).

CHD = coronary heart disease; HDL = high-density lipoprotein; LDL = low-density lipoprotein; CAC = coronary artery calcium score. Source: Authors’ calculations from the Multi-Ethnic Study of Atherosclerosis.
