# Supplementary material for: Cost-Effectiveness of Coronary Artery Calcium Testing for Coronary Heart and Cardiovascular Disease Risk Prediction to Guide Statin Allocation: The Multi-Ethnic Study of Atherosclerosis (MESA)
Source: PLoS One. 2015 Mar 18;10(3):e0116377. doi: 10.1371/journal.pone.0116377 (PMC4364761; doi:10.1371/journal.pone.0116377)
Supplement: S2 Table — Note: The results presented are mean costs and effects calculated over 1,000 simulations. Negative numbers in the mean effects columns are counts of events or losses of QALYs (depending on the valuation of outcomes). Italicized lines indicate that only patients with CAC≥100 are treated in the CAC strategy. ICER = Incremental Cost-Effectiveness Ratio. The model scenario numbers correspond to the scenarios presented in Table 4. (DOCX) [file pone.0116377.s003.docx]

S2 Table: Results Using Base-Case MESA Event Rates

| Scenario | Valuation of Outcomes | Treat All Mean Cost ($) | Treat All Mean Effect | ATP III Mean Cost ($) | ATP III Mean Effect | CAC Mean Cost ($) | CAC Mean Effect | Decision |
| --- | --- | --- | --- | --- | --- | --- | --- | --- |
| CHD Events | | | | | | | | |
| 1 | Events | 2093.561 | -0.02788 | 2114.508 | -0.03174 | 1993.857 | -0.02665 | CAC Dominates Both |
| 2 | QALYs | 2093.561 | -0.01555 | 2114.508 | -0.01299 | 1993.857 | -0.01287 | CAC Dominates Both |
| ***3*** | *Events* | *2093.561* | *-0.02788* | *2114.508* | *-0.03174* | *2024.142* | *-0.02904* | *Treat All Dominates ATP III* |
| ***4*** | *QALYs* | *2093.561* | *-0.01555* | *2114.508* | *-0.01299* | *2024.142* | *-0.0115* | *CAC Dominates Both* |
| 5 | Events | 3546.421 | -0.04611 | 3519.222 | -0.05233 | 3304.644 | -0.04448 | CAC Dominates Both |
| 6 | QALYs | 3546.421 | -0.04114 | 3519.222 | -0.03805 | 3304.644 | -0.03613 | CAC Dominates Both |
| ***7*** | *Events* | *3546.421* | *-0.04611* | *3519.222* | *-0.05233* | *3301.945* | *-0.04807* | *Treat All Cost-Effective; ICER=$4,373* |
| ***8*** | *QALYs* | *3546.421* | *-0.04114* | *3519.222* | *-0.03805* | *3301.945* | *-0.03457* | *CAC Dominates* |
| Sensitivity Analyses on Cost Parameters | | | | | | | | |
| 9 | Events | 1852.289 | -0.02788 | 2022.94 | -0.03174 | 1840.434 | -0.02665 | CAC Dominates Both |
| 10 | QALYs | 1852.289 | -0.01557 | 2022.94 | -0.01294 | 1840.434 | -0.01287 | CAC Dominates Both |
| 11 | QALYs | 3615.426 | -0.01557 | 2692.091 | -0.01294 | 2961.599 | -0.01287 | ATP III (Status Quo) |
| ***12*** | *QALYs* | *3615.005* | *-0.01557* | *2703.015* | *-0.01294* | *2484.982* | *-0.0115* | *CAC Dominates Both* |
| 13 | QALYs | 2093.561 | -0.01557 | 2114.508 | -0.01294 | 1968.857 | -0.01287 | CAC Dominates Both |
| ***14*** | *QALYs* | *2093.561* | *-0.01557* | *2114.508* | *-0.01294* | *1999.143* | *-0.0115* | *CAC Dominates Both* |
| 15 | QALYs | 2093.561 | -0.01557 | 2114.508 | -0.01294 | 2043.857 | -0.01287 | CAC Dominates Both |
| ***16*** | *QALYs* | *2093.561* | *-0.01557* | *2114.508* | *-0.01294* | *2074.143* | *-0.0115* | *CAC Dominates Both* |
| 17 | QALYs | 2093.561 | -0.01557 | 2114.508 | -0.01294 | 2143.857 | -0.01287 | ATP III (Status Quo) |
| 18 | QALYs | 2124.135 | -0.01557 | 2149.257 | -0.01294 | 2057.054 | -0.01287 | CAC Dominates Both |
| CVD Events | | | | | | | | |
| 19 | Events | 2282.603 | -0.03573 | 2337.142 | -0.04084 | 2218.219 | -0.03501 | CAC Dominates Both |
| 20 | QALYs | 2282.603 | -0.01851 | 2337.142 | -0.01647 | 2218.219 | -0.01609 | CAC Dominates Both |
| ***21*** | *Events* | *2282.603* | *-0.03573* | *2337.142* | *-0.04084* | *2267.854* | *-0.03802* | *Treat All Dominates ATP III* |
| ***22*** | *QALYs* | *2282.603* | *-0.01851* | *2337.142* | *-0.01647* | *2267.854* | *-0.01494* | *CAC Dominates Both* |
| 23 | Events | 4121.711 | -0.06401 | 4174.105 | -0.07265 | 3922.577 | -0.0629 | CAC Dominates Both |
| 24 | QALYs | 4121.711 | -0.05306 | 4174.105 | -0.05165 | 3922.577 | -0.04908 | CAC Dominates Both |
| ***25*** | *Events* | *4121.711* | *-0.06401* | *4174.105* | *-0.07265* | *3981.108* | *-0.06802* | *Treat All Dominates ATP III* |
| ***26*** | *QALYs* | *4121.711* | *-0.05306* | *4174.105* | *-0.05165* | *3981.108* | *-0.0488* | *CAC Dominates Both* |
| Sensitivity Analyses on Cost Parameters | | | | | | | | |
| 27 | Events | 2042.262 | -0.03573 | 2246.071 | -0.04084 | 2065.292 | -0.03501 | CAC Dominates ATP III |
| 28 | QALYs | 2042.262 | -0.01851 | 2246.071 | -0.01647 | 2065.292 | -0.01609 | CAC Dominates ATP III |
| 29 | QALYs | 3798.597 | -0.01851 | 2911.59 | -0.01647 | 3182.838 | -0.01609 | ATP III (Status Quo) |
| ***30*** | *QALYs* | *3798.597* | *-0.01851* | *2911.59* | *-0.01647* | *2726.634* | *-0.01494* | *CAC Dominates Both* |
| 31 | QALYs | 2282.603 | -0.01851 | 2337.142 | -0.01647 | 2193.219 | -0.01609 | CAC Dominates Both |
| ***32*** | *QALYs* | *2282.603* | *-0.01851* | *2337.142* | *-0.01647* | *2243.854* | *-0.01494* | *CAC Dominates Both* |
| 33 | QALYs | 2282.603 | -0.01851 | 2337.142 | -0.01647 | 2268.219 | -0.01609 | CAC Dominates Both |
| ***34*** | *QALYs* | *2282.603* | *-0.01851* | *2337.142* | *-0.01647* | *2317.854* | *-0.01494* | *CAC Dominates ATP III* |
| 35 | QALYs | 2282.603 | -0.01851 | 2337.142 | -0.01647 | 2368.217 | -0.01609 | ATP III (Status Quo) |
| 36 | QALYs | 2329.571 | -0.01851 | 2390.841 | -0.01647 | 2298.05 | -0.01609 | CAC Dominates Both |

Note: The results presented are mean costs and effects calculated over 1,000 simulations. Negative numbers in the mean effects columns are counts of events or losses of QALYs (depending on the valuation of outcomes). Italicized lines indicate that only patients with CAC≥100 are treated in the CAC strategy. ICER=Incremental Cost-Effectiveness Ratio. The model scenario numbers correspond to the scenarios presented in Table 4.
